# Rhinosporidiosis of the lacrimal sac masquerading as chronic dacryocystitis: a rare presentation

**DOI:** 10.4322/acr.2020.214

**Published:** 2021-01-20

**Authors:** Pradeep Pradhan, Swagatika Samal

**Affiliations:** 1 All India Institute of Medical Sciences, Department of ENT and Head Neck Surgery, Bhubaneswar, Odisha, India; 2 All India Institute of Medical Sciences, Department of Pathology, Bhubaneswar, Odisha, India

**Keywords:** Lacrimal Apparatus Diseases, Eye Diseases, Rhinosporidiosis, Mesomycetozoea infections, Endemic Diseases

## Abstract

Rhinosporidiosis is a chronic infection of the mucous membrane caused by the *Rhinosporiduim seeberi*, which infects through transepithelial penetration. Although described worldwide, this entity is mostly found in the western hemisphere, afflicting young people, predominantly males, associated in many cases with recreational or professional contact with bath in ponds, rivers, or stagnant waters. The clinical features are varied depending on the affected membrane, in some cases mimicking other diseases postponing the correct diagnosis. Although nasal obstruction and epistaxis are the common clinical presentations in sinonasal rhinosporidiosis, patients with epiphora without a nasal mass often challenge the diagnosis. In the present case, we have documented a case of isolated lacrimal sac rhinosporidiosis masquerading as chronic dacryocystitis, which was successfully managed by endoscopic excision, accompanied by a literature review.

## INTRODUCTION

Rhinosporidiosis is a chronic granulomatous disease caused by *Rhinosporidium seeberi*, frequently encountered in the southern zone of India and Sri Lanka.^1^ The nose and nasopharynx are the most common site affected by the disease and patients usually present with a painless mass with a history of nasal bleeding.^2^ It often presents as a polypoidal mass in the nasal cavity^3^. Although nasal obstruction and epistaxis are the common clinical features, the epiphora as the single symptom is a challenging diagnosis. In the present case, we have documented a case of isolated lacrimal sac rhinosporidiosis masquerading as chronic dacryocystitis, which was successfully managed by endoscopic excision.

## CASE REPORT

**Figure 1:**
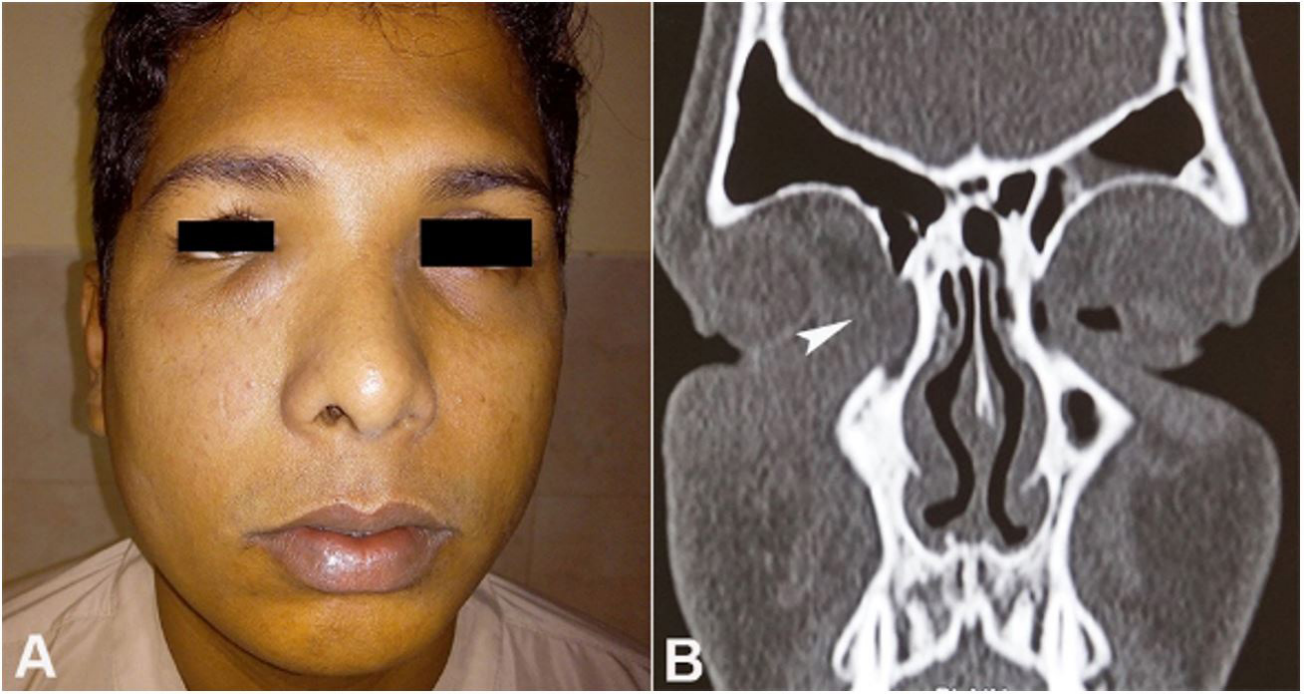
**A** – External view of the patient’s face showing the **s**welling below the medial canthus of the right eye; **B** – Non-contrast CT scan (Coronal plane) revealed a soft tissue density in the lacrimal sac without bony erosion (arrowhead).

A 25-year-male patient presented to the outpatient clinic with a swelling below the medial canthus of the right eye involving the lower eyelid for 12 months (Figure 1A). A history of epiphora was present for the last six months. There was no history of nasal obstruction or epistaxis in the past. Anterior rhinoscopy did not reveal any abnormality in the nasal cavity. Nasal patency was found to be equal in both nasal cavities. A non-contrast CT scan was suggestive of a soft tissue density in the lacrimal sac region without any erosion of the lacrimal bone (Figure 1B).

**Figure 2:**
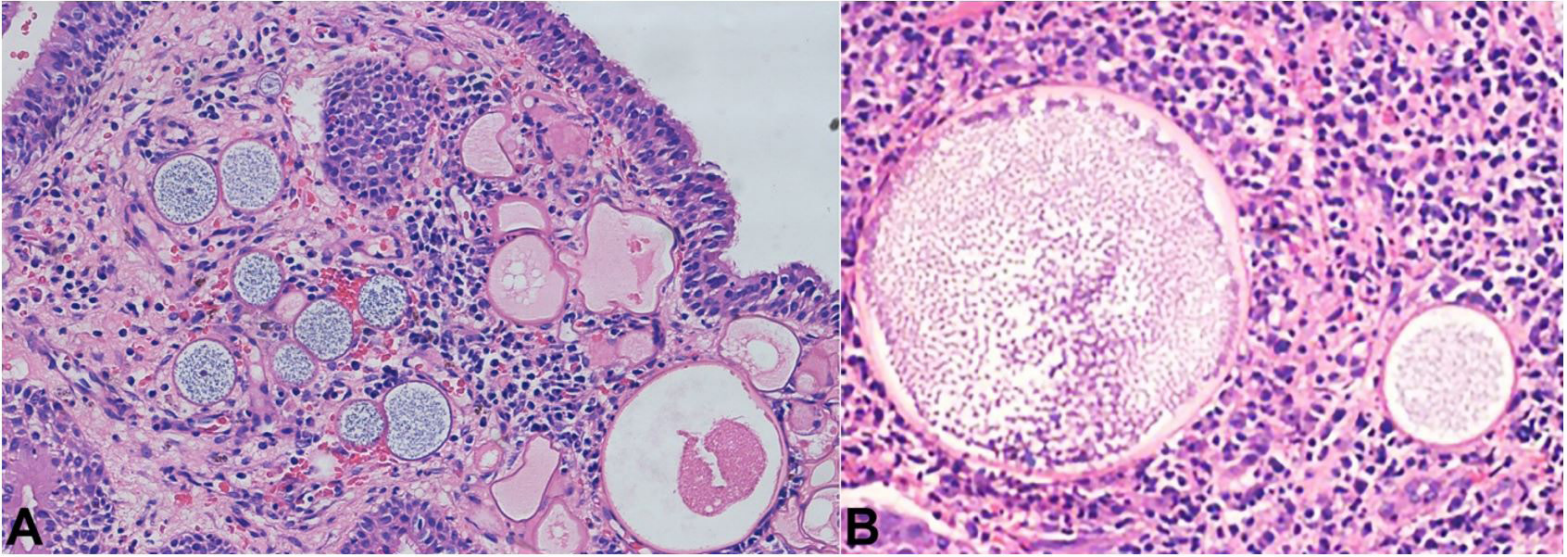
Photomicrograph of the surgical specimen. **A** – showing globular cysts representing the sporangia containing daughter spores in different stages of development (H&E 100x); **B** – Shows sporangia which are surrounded by dense chronic inflammatory infiltrate comprising of lymphocytes and plasma cells (H&E 200x).

The visual acuity was normal in both eyes. Syringing the right lacrimal passage revealed a hard stop, consistent with the obstruction of the right nasolacrimal duct. After the informed written consent, the patient was planned for endoscopic dacryocystorhinostomy (DCR). After drilling out of the ascending process of maxilla, a dilated lacrimal sac was detected. On incising the sac, a polypoidal bleeding mass was detected obliterating the whole lumen. The mass was completely excised after a gentle dissection of the sac wall. Endoscopic dacryocystorhinostomy was performed as an adjunctive procedure to maintain the lacrimal patency. The histopathology of the specimen was confirmed to be rhinosporidiosis (Figure 2).

The patient is on regular follow in the rhinology clinic and the ophthalmology department for the past 12 months without any recurrence of the disease.

## DISCUSSION

Rhinosporidiosis is a chronic granulomatous disease affecting both humans and animals, caused by *Rhinosporidium seeberi*. Although it was first classified under fungal disease under ICD10, it is now considered an aquatic protistan parasite belonging to the class Mesomycetozoa^4^. The fish and the aquatic amphibians are considered the natural host of the parasite, Humans acquire the disease by accidental contact or bathing in ponds, rivers, or working in stagnant water.^5^ Transmission from animals to humans often occurs through water contact. Although already encountered worldwide, it notably endemic in the Southern part of India and Sri Lanka.^6^ Rhinosporidiosis was first identified in 1892, but comprehensively described in 1900 by Guillermo Seeber in a farm-worker in Argentina with impaired breathing by a nasal mass. Kuriakose^7^ first described the ocular rhinosporidiosis in 1963. The nose and nasopharynx are the primary sites to be affected by the infection (78%), followed by the conjunctiva (15%).^5^ The lacrimal system becomes affected in 14.3% to 59.6% of cases of ocular rhinosporidiosis.^8^ ^-^ ^11^ The ocular involvement is mostly presented by a conjunctival polyp (77.6%), either in the upper or lower lid of the fornix. Approximately 26% of the cases of ocular rhinosporidiosis are associated with either nasal or conjunctival involvement.^12^ Isolated lacrimal sac rhinosporidiosis is very rare in the clinical practice with only a few reports in the literature.^9^ ^,^ ^10^ It is always a challenge to suspect a case of isolated lacrimal sac rhinosporidiosis as the clinical feature always resembles a chronic dacryocystitis. Radiological evaluation (CT scan /MRI) can be undertaken in selective cases to rule out a similar pathology.^13^ We undertook a literature review based on the PubMed database using “rhinosporidiosis and lacrimal sac” as the keywords. A total of 29 articles were retrieved, which comprised publications from 1973 to 2019. 156 cases of lacrimal sac rhinosporidiosis have been reported and are summarized in Table 1, including the predominant symptoms, the primary site of involvement, and the various treatment protocols. From the 156 cases, the main symptom was lacrimal sac swelling, which was observed in almost all cases. Epiphora was seen in 10.25% of the cases. In our case, epiphora and lacrimal swelling were the only presenting complaints that misled the working diagnosis toward a dacryocystitis/dacryocystocele. Epiphora can be continuous,^4^ ^,^ ^17^ ^,^ ^32^ intermittent,^18^ ^,^ ^23^ ^,^ ^29^ ^,^ ^33^ bloody^24^ ^,^ ^27^, mucopurulent,^18^ ^,^ ^34^ or sometimes it is absent.^5^ ^,^ ^15^ ^,^ ^19^ ^,^ ^20^ ^,^ ^25^ ^,^ ^36^ ^,^ ^38^ The absence of lacrimal swelling could be due to the spread of infection through the peri canalicular space of the lacrimal sac without affecting the drainage pathway.^18^ ^,^ ^39^ The etiological agent can reach the lacrimal sac from the nose or conjunctiva,^40^ unlike the present case where the mass was found only in the lacrimal sac, without a synchronous nasal involvement. Due to the complete obstruction of the lacrimal flow, the patient presented with epiphora resembling a chronic dacryocystitis. There are various schools of thought on the possible route by which rhinosporidiosis involves the lacrimal sac. Some researchers think it spreads through the nasolacrimal duct to the lacrimal sac and others comment that it does not spread through the duct because of the presence of the valve.^16^ The imaging examination of the nose and paranasal sinus is considered a key resource to diagnose patients with isolated rhinosporidiosis, as it is often mistaken with chronic dacryocystitis. On computed tomography, it appears as a homogenously enhanced mass in the inferior meatus extending to the lacrimal sac with the erosion of the adjacent bone.^41^ However, in the present case, the mass was only limited to the lacrimal sac without any radiological evidence of bony erosion. Although the majority of patients with sinonasal rhinosporidiosis are diagnosed on the clinical findings, histopathology is warranted for its confirmation.^42^ On histopathology, the lesion presents with a distinctive morphology, consisting of globular cysts representing the thick-walled sporangia containing more than 1000 daughter spores in different stages of development, accompanied by a mixed inflammatory infiltrate. The organism is stained by periodic acid Schiff (PAS), Gomori’s methenamine silver and Mucicarmine.^22^

**Table 1:** Shows the review of literature of the lacrimal sac rhinosporidiosis

Author	No of cases	Presentation	Epiphora	Site	Treatment	Follow-up months	Relapse	Medical therapy
Gupta et al.^14^	1	Rt medial canthal swelling extending nasal bridge	Present	LS, NLD & Nose	Endoscopic dacryocystorhinostomy	6	NM	PVP-I 2 minutes
Suneer and Sivasankari ^15^	2	NM	Nil	LS & NLD	NM	NM	NM	NM
Prabhu et al.^16^	4	NM	NM	LS, NLD & N	LS was excised, pink vacuolization polypoidal growth	NM	NM	NM
Rajesh Raju and Sandeep^17^	13	Swelling over LS area, Blood stained nasal discharge	in 2 cases	LS, NLD & N	Endoscopic DCR with NLD excision	16	1/13	NM
Girish and Prathima^18^	1	Diffuse nontender infra orbital swelling of the left eye	Intermittent	NM	DCT with “en bloc” resection of NLD.	NM	NM	DDS100 mg OD for 3 months
Chakraborti et al.^19^	1	Painless swelling of left lower eyelid	Nil	LS	orbitotomy - sub ciliary approach, diverticula were removed leaving sac behind. Recurred with fistula	12	Noted after 12 months	PVP-I +Amocla
Jamison et al.^20^	1	Swelling at nasal aspect of left lower lid.	Nil	LS, NLD & N	Gelatinous lesion attached to superior wall of lacrimal sac extending into NLD.	5	NM	NM
Basu et al.^12^	1	Pinkish swelling over left lower orbital area for 3 years	Nil	LS & NLD	DCT, excision of mass with sac	6	Nil	DDS 100 mg/d OD 6 m
Mishra et al.^21^	1	Swelling at the medial canthus of left eye	Present	LS + NLD	incision over the medial canthus of left eye. Mass with sac removed. Silastic tube placed from punctum to nose	1	NIL	DDS 100mg OD X1 months
Nuruddin et al.^22^	18	Swelling in LS area, epistaxis and blood-stained discharge	NM	LS =16, fistula= 2	Modified DCR. A small portion of the sac around common canaliculi was left, DCR tube placed	12	2/18	PVP-I for 2 minutes
Sah^23^	1	Left medial infraorbital diffuse nontender swelling	Intermittent	LS & NLD	Multiple tiny vascularized growth. Sac was sutured and removed “en bloc” with NLD	24	Nil	DDS 100 mg/d 3m
Guru and Pradhan^24^	10	Blood- tinged discharge from eye	Blood tinged discharge	LS & N n=7, NLD & N n=3	DCT, cauterisation of base. Debridement of mucous membrane of NLD	NM	NM	NM
Mukherjee et al.^25^	1	Recurrent painful swelling below right lower lid	Nil	LS	DCT with wide excision with cauterization	NM	NM	PVP-I & DDS 100 mg OD 6 m
Billiveau et al.^26^	1	Bloody mucopurulent tear, swelling in the medial canthal area of the left eye	Bloody tear	LS & NLD	Open excision & biopsy, followed by external DCR	60	NIL	NM
Mithal et al.^27^	13	mucocele=4, swelling= 3	Blood tinged discharge= 2	LS	NM	Mean 14,2	1	NM
Pusker et al.^8^	1	Swelling Rt medial canthal area & Mucopurulent discharge	Present	LS & NLD	DCT & “en bloc” excision of the extension growth in the nasopharynx	6	NIL	DDS 100 mg OD 6 months
Rogers et al.^28^	1	Swelling of the left inner canthus.	Present	LS	External DCR	120	Yes	Nill
Ghosh et al.^29^	1	Swelling in medial canthus area, epiphora and purulent discharge from eye.	Present	LS	Endoscopic DCR + DCT	NM	NM	NM
Varshney et al.^30^	1	Right facial swelling, nasal obstruction and intermittent nasal bleeding	Present	LS, N & O	Lateral rhinotomy with sac excision by parapharyngeal route	3	Nil	NM
Ghorpade et al.^31^	1	Swelling under right eye with scanty bloody nasal discharge.	NM	LS, NLD & N	Naso-optic sulcus incision. Mass filled the lacrimal sac and lacrimal duct, separately excised followed by electrocautery	8	NIL	DDS 100 mg daily, length- NM
Chowdhury et al.^10^	3	Epistaxis	NM	LS n = 1, LS & SC n = 2	DCT done, pink vascularized finger like extension was seen in all the cases	12	SC spread n=2	NM
Watve et al.^32^	1	swelling Rt medial canthal area	Present & purulent discharge	LS	Endoscopic DCR, a mass popped out of the sac	NM	Nil	DDS 100 mg alternate day 12 m
Nerurkar et al.^33^	1	Diffuse, soft, non- tender Rt infraorbital swelling. diffuse	Intermittent	LS	Endoscopic DCR	<1	Present	DDS 50 mg/d -3 months
Thakur et al.^34^	3	Purulent discharge=2, swelling lower fornix 2	Present=3	LS	DCT	NM	NM	PVP-I = 1
Krishnan et al.^35^	1	Swelling at the inner canthus of the right eye, occasional blood- stained discharge	Present	LS	DCT (sac + diverticula)	NM	NM	NM
Mukharjee et al.^14^	48	LS diffuse swelling n=45; nose bridge widening n = 42; lower lid swelling n = 30; LS localized swelling n = 3, nose bleeding= 6	Nil	LS n=42, LS & N n=6	DCT	NM	Nil	NM
Suseela and Subramaniam^36^	7	Epistaxis as the lesions involved nose,	Nil	LS & nose	Excision biopsy	5 out of 7	NM	Yes
David and Sivaramasubrahmanyam^37^	21	Growth between lids and globe, Swelling lower lid	Nasal bloody discharge	LS + Nose & Limbus	DCT and excision of conjunctival growths	NM	1	NM

Amocla= amoxicillin + clavulanate, DCR: Dacryocystorhinostomy, DCT: Dacryocystectomy, DDS: Dapsone, IM: Inferior meatus, IT: Inferior turbinate, LS: Lacrimal sac, Lt: Left, n: Number of cases, N= nose, NLD: Nasolacrimal duct, NM: Not mentioned, m: month, O: oropharynx, OD: Once daily, PVP-I: Povidone-Iodine, Rt: Right, SC: subcutaneous.

Surgical excision with electrocautery of the base of the lesion is considered the treatment of choice for patients with sinonasal rhinosporidiosis. The majority of the patients are managed through external dacryocystorhinostomy.^21^ ^,^ ^26^ ^,^ ^28^ ^,^ ^30^ ^,^ ^31^ ^,^ ^35^ ^,^ ^37^ In contrast, in the present case, the mass was excised through a transnasal endoscopic procedure, similar to the standard endoscopic dacryocystorhinostomy, as described by Gupta et al.^14^ Although medical treatment cannot be denied, its role is very much limited to patients where complete surgical resection is not possible due to the systemic spread of the disease. Dapsone, because of its anti rhinosporidial activity, can be used to arrest the maturation of sporangia and to promote fibrosis in the stroma. It can be used with 100 mg once/twice daily for 3-6 months in the preoperative period to prevent the recurrence.^4^ ^,^ ^18^ ^,^ ^23^ ^,^ ^24^ ^,^ ^29^ Due to the advancement of the transnasal endoscopic procedures, there has been a decrease in the disease’s recurrence rate due to enhanced visualization ensuring complete removal of the disease, as observed in the present case. Dapsone was not prescribed in our present as the disease was only limited to a lacrimal sac where complete removal was ensured with the use of wide-angle rigid nasal endoscopes. Despite the low recurrence rate, each patient needs a close follow-up with routine nasal endoscopy and lacrimal patency tests to find out the early recurrence of the disease. A high index of suspicion of the disease, appropriate radiological examination, and histopathology are considered the cornerstones for the diagnosis of an isolated rhinosporidiosis of the lacrimal sac. As described in the present case, isolated rhinosporidiosis of the lacrimal sac can be a potential differential diagnosis of chronic dacryocystitis, especially with atypical presentation.

## CONCLUSION

Although patients with ocular rhinosporidiosis are mostly diagnosed on the clinical findings, i.e., with nasal obstruction and epistaxis, it is often a challenge to diagnose a case where epiphora is the single complaint. In combination with the radiological and histopathological examination, a proper clinical history is mandatory for the accurate diagnosis of atypical rhinosporidiosis.

All procedures performed in studies involving human participants were in accordance with the ethical standards of the institutional and/or National research committee and with the 1964 Helsinki declaration and its later amendments or comparable ethical standards. Written informed consent has been taken from the patient prior to surgery and has been informed to the Institute reviewer board.
